# Microelectrode Arrays Measure Blocking of Voltage‐Gated Calcium Ion Channels on Supported Lipid Bilayers Derived from Primary Neurons

**DOI:** 10.1002/advs.202304301

**Published:** 2023-12-01

**Authors:** Zixuan Lu, Chiara Barberio, Ana Fernandez‐Villegas, Aimee Withers, Alexandra Wheeler, Konstantinos Kallitsis, Eleonora Martinelli, Achilleas Savva, Becky M. Hess, Anna‐Maria Pappa, Gabriele S. Kaminski Schierle, Róisín M. Owens

**Affiliations:** ^1^ Department of Chemical Engineering and Biotechnology University of Cambridge Philippa Fawcett Drive Cambridge CB3 0AS UK; ^2^ Pacific Northwest National Laboratory 902 Battelle Boulevard Richland WA 99 354 USA; ^3^ Department of Biomedical Engineering Khalifa University of Science and Technology Abu Dhabi 127788 UAE; ^4^ Healthcare Engineering Innovation Center (HEIC) Khalifa University of Science and Technology Abu Dhabi 127 788 UAE

**Keywords:** blebbing, microelectrode arrays, neurons, PEDOT:PSS, supported lipid bilayers, voltage‐gated ion channels

## Abstract

Drug studies targeting neuronal ion channels are crucial to understand neuronal function and develop therapies for neurological diseases. The traditional method to study neuronal ion‐channel activities heavily relies on the whole‐cell patch clamp as the industry standard. However, this technique is both technically challenging and labour‐intensive, while involving the complexity of keeping cells alive with low throughput. Therefore, the shortcomings are limiting the efficiency of ion‐channel‐related neuroscience research and drug testing. Here, this work reports a new system of integrating neuron membranes with organic microelectrode arrays (OMEAs) for ion‐channel‐related drug studies. This work demonstrates that the supported lipid bilayers (SLBs) derived from both neuron‐like (neuroblastoma) cells and primary neurons are integrated with OMEAs for the first time. The increased expression of voltage‐gated calcium (CaV) ion channels on differentiated SH‐SY5Y SLBs  compared to non‐differentiated ones is sensed electrically. Also, dose‐response of the CaV ion‐channel blocking effect on primary cortical neuronal SLBs from rats is monitored. The dose range causing ion channel blocking is comparable to literature. This system overcomes the major challenges from traditional methods (e.g., patch clamp) and showcases an easy‐to‐test, rapid, ultra‐sensitive, cell‐free, and high‐throughput platform to monitor dose‐dependent ion‐channel blocking effects on native neuronal membranes.

## Graphical abstract

Primary neuron membranes can be isolated and integrated with microelectrode arrays. This is a study using electrochemical impedance spectroscopy for monitoring the neuronal membrane quality and detecting ion channel blockage on the neuron membranes, which could be a novel technique for drug screening and neuroscience research.

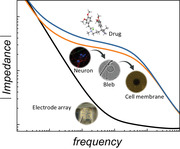

## 1 Introduction

Since Hodgkin and Huxley first measured the voltage‐gated ion channel current from a giant squid axon using the patch clamp method,^[^ ^1^ ^]^ numerous studies of ion‐channel function using whole‐cell patch clamp followed, rendering it one of the most important techniques to study neuronal activity and related diseases.^[^ ^2^ ^]^ Despite being information‐rich and accurate, conventional patch clamp assays rely on contacting the cell surface with a pipette to monitor the membrane of a single cell which severely limits the recording output, both in terms of duration and in terms of yield (i.e., the number of examined samples). Additional drawbacks include the need for sterility and maintenance of the highly controlled cell‐culture conditions, the limited sampling area (tip of the nano/micropipette) on the cell membrane, and the practical limitations to extend to high‐throughput and multiplexing systems.^[^ ^3^, ^4^ ^]^ To overcome these shortcomings, planar patch clamp, as an alternative, was developed, and is now commercially available through products such as Syncropatch 384 by Nanion Technologies, Qpatch by Sophion Bioscience, IonFlux Mercury by Fluxion Biosciences, and others.^[^ ^5^ ^]^ Cells are placed and cultured on a surface with patch clamp apertures, so that the electronic system can sense the ionic flow through the ion‐channels via the apertures.^[^ ^6^ ^]^ This technique has been adapted with multi‐well culture and robotic pipetting systems, to realise high‐throughput and automated systems. However, isolated primary neurons, once cultured on the apertures of a patch clamp chip, do not extend neurites and do not have the complex growth patterns seen on standardised culture substrates.^[^ ^7^ ^]^ Another alternative, microelectrode arrays (MEAs) with neuronal cells cultivated on top, can measure populations of neurons, local field potential (LFP), but cannot measure intracellular ion channel activity to date.^[^ ^8^ ^]^ A lot of work has been done trying to get 3D electrodes to approach cells with a smaller than 50 nm cleft to be able to achieve intracellular recordings, necessary to record ion channel function.^[^ ^7^, ^9^, ^10^ ^]^

For whole‐cell recording by MEAs or planar patch clamp, cells must be cultivated on the surface where they are recorded. In the case of neurons, it is impossible to transfer them once differentiated or matured. Ideally, cells can be cultivated on an optimal surface (e.g., standard culture flasks), and then investigated electrically. We have previously shown the “biomembrane‐on‐a‐chip” concept, applied to interface cell‐derived/ or mimicking membranes in the form of lipid bilayers with an electronic transducer allowing for electronic monitoring of membrane related events, including ion‐channel activity, drug‐protein/lipid interactions, and virus‐membrane fusion events.^[^ ^11^, ^12^, ^13^, ^14^, ^15^ ^]^ We showed the ability to measure ion channel activity from supported lipid bilayers created from blebs derived from cell lines. Specifically, opening of ATP‐gated ion‐channels was detected upon addition of ATP, and both opening and closing of TREK‐1 ion‐channels was sensed when exposed to TREK‐1 blockers/openers.^[^ ^11^, ^12^ ^]^

There are a number of benefits to the use of the biomembrane‐on‐a‐chip platform. One is that the complexity of the supported lipid bilayers (SLBs) can be finely tuned, ranging from simple lipid‐only layers^[^ ^13^, ^14^, ^16^ ^]^ to cell‐derived proteinaceous SLBs displaying all the complexity of the cell membrane.^[^ ^11^, ^12^, ^17^ ^]^ Typically, SLBs are formed on flat substrates such as glass or mica.^[^ ^18^, ^19^ ^]^ Then, microscopy techniques, such as transmission electron microscopy (TEM), atomic force microscopy (AFM), and Raman spectroscopy can be applied to characterize structural and topographical properties of native neuronal membranes and ion channels on these flat substrates.^[^ ^20^ ^]^ However, these flat substrates cause the lack of “fluidity” beneath the bilayer and the inability of the proteins to extend below the bilayer structure posing limitations in terms of their mobility and function.^[^ ^21^ ^]^ The same applies for most electroactive substrates used to date to interface electrical models of cell membranes.^[^ ^4^ ^]^ This is typically improved by adding hydrogel‐like materials between the substrate and the bilayer to provide a more fluid and ECM‐mimicking environment. To overcome this challenge, a substantial benefit of our platform lies in the use of organic mixed ionic/electronic conductors (OMIECs), such as the well‐known poly (3,4‐ethylenedioxythiophene) polystyrene sulfonate (PEDOT:PSS), combining both electrical activity and tissue‐matching mechanical properties. These materials have been shown to provide an ideal interface for SLB formation and protein function (i.e., orientation and mobility) as confirmed by advanced surface characterisation methods.^[^ ^22^ ^]^ Another benefit of using PEDOT:PSS‐based devices for such purposes is the optical transparency of the OMIEC films that allows for optical assessment in conjunction with the electrical assessment. Electrochemical impedance spectroscopy (EIS) is usually employed with PEDOT:PSS devices for recording SLB barrier properties in the form of frequency‐dependent impedance spectra, which consist of resistive and capacitive properties of SLBs.^[^ ^17^ ^]^ EIS measurements can be presented as Bode plots (impedance versus frequency), which demonstrate the net impedance of the whole system. The impedance features of SLB are generally a “slope” at high frequencies and a plateau at mid‐frequencies, which represent the membrane capacitance and resistance, respectively.^[^ ^17^, ^22^ ^]^ Also, the same EIS data of SLBs can be presented as Nyquist plots, which plot the real part (having information of membrane resistance) versus the imaginary part (having information membrane capacitance) of the membrane. By decoupling the changes in resistance and capacitance of an SLB, the exact biological event occurring on the SLB can be depicted. Generally, ion channels function as resistors, which provide pathways for ions to be transported through the membrane. Typically, open ion channels would result in a decrease in SLB resistance, whereas closed ion channels cause an increasing in SLB resistance.^[^ ^17^ ^]^

To date, the biomembrane‐on‐a‐chip platform has not been demonstrated for use in ion channel monitoring of primary cells, specifically neurons. Our previous work on ion channels (TREK1, P2×2) was based on the overexpression of the protein of interest within cell lines (e.g., HEK 293 cells) to achieve specific monitoring of protein activity/interactions. Although highly sensitive responses were obtained, thanks to by the abundance of the transmembrane proteins of interest on the SLB surfaces, the need to overexpress the receptor of choice to perform a given experiment limits its practicality and strays from the in vivo context. Being able to monitor events at the cell membranes on naturally abundant cell membrane proteins remains a challenge which represents the core of the current study, focused on characterising ion channels present in differentiated neuronal cells, specifically a voltage‐gated calcium ion channel.

Generally, voltage‐gated ion channels play a crucial role in cell signaling. Amongst them, voltage‐gated calcium (CaV) ion channels directly participate in key physiological events such as, cardiomyocyte contraction, release of hormones, and neurontransmitters.^[^ ^23^ ^]^ In a neuron, a typical ionic current through a CaV ion channel is one of the most important signals for interneuronal communication.^[^ ^24^ ^]^ Influx of Ca^2+^ causes fusion of synaptic vesicles and release of neurotransmitters into the synaptic cleft to trigger the propagation of action potential. Dysfunction of CaV ion‐channels has been found to cause several neurological disorders,^[^ ^25^ ^]^ such as epilepsy, neuropathic pain, Parkinson's disease, and many more.^[^ ^23^, ^25^, ^26^, ^27^ ^]^ A number of molecules targeting CaV ion channels work as ion‐channel blockers in order to reduce the trafficking across the cell membrane to regulate or reduce the severity of symptoms.^[^ ^25^ ^]^ Based on the specificity of the blocking mechanism of CaV ion channels, drug molecules interact with the interior binding sites of CaV ion channels as targets to trigger the closing mechanism as a direct route to develop a new generation of drugs. Novel screening platforms are required to determine drug efficacy and to resolve the neurological disease burden related to CaV ion‐channel dysfunctions.

In neuroscience research, primary animal neuron cultures represent the most reliable in vitro models and cell types in terms of biological significance, to mimic human neuronal functions. However, animal dissection for primary neuronal cell isolation and post‐experimental euthanasia inevitably pose ethical concerns on top of challenges in terms of practical applicability.^[^ ^28^ ^]^ Also, once the primary neurons are cultured to mature neurons, they lose their ability to proliferate, which limits the reusability of the culture and increases the demands for animal dissection. Despite these issues, primary neuron harvesting and culture remain the gold standard for studying electrophysiological activities in neuroscience in vitro. On the other hand, neuron‐like immortalized cell lines, such as neuroblastoma lines, are compatible with cryopreservation and stable during multiple passages.^[^ ^29^ ^]^ As such, SH‐SY5Y neuroblastoma cells represent a widely explored cell line for in vitro neuroscience research. Further, SH‐SY5Y cells can be differentiated to a more mature neuron‐like phenotype expressing mature neuronal markers via continuous treatments with retinoic acid (RA) and brain‐derived neurotrophic factor (BDNF).^[^ ^29^, ^30^, ^31^ ^]^ In terms of CaV ion‐channels, SH‐SY5Y cells naturally express multiple Cav ion‐channel proteins, including three main subtypes: CaV1.3, CaV2.2, and CaV3.3.^[^ ^23^ ^]^ After differentiation with RA, Toselli and colleagues, using the patch clamp method, showed evidence of higher ionic calcium current through SH‐SY5Y CaV ion‐channels, one of the hallmarks for a matured neuron.^[^ ^32^ ^]^

In this work, we first performed continuous differentiation of SH‐SY5Y neuroblastoma with RA and BDNF. Then, the vesiculation process and characteristics of the native vesicles (blebs) were studied for the SH‐SY5Y cells before and after differentiation. We demonstrate the formation of a reconstituted cell membrane from these cells on PEDOT:PSS microelectrode arrays, and successfully sense upregulated dose‐response blocking effects of naturally expressed CaV ion channels after SH‐SY5Y differentiation, with EIS. Further, to confirm whether the CaV ion‐channel expression level of the differentiated SH‐SY5Y membrane is comparable to primary neuron membranes, we showed the bleb harvesting and formation of native membranes on PEDOT:PSS microelectrode arrays from rat cortical neurons, for the first time. The response of the CaV ion‐channel blocking effect from the cortical neuron membrane is comparable to the enhanced expression of CaV ion channels after SH‐SY5Y differentiation after applying the same dose‐response experiments. This work demonstrates the ability to use our native‐membrane‐on‐a‐chip platform as a tool for studying drug interaction with neuronal membrane proteins at their natural concentrations and without the need for pre‐engineering of the cells to overexpress the receptors of interest. Our platform thus represents a rapid, scalable and cell‐free alternative to patch clamp.

## 2 Results

**Figure 1:**
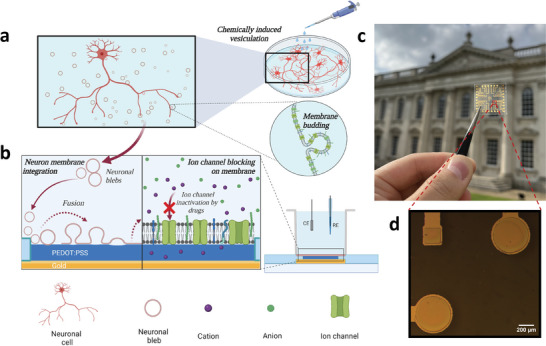
Neuronal membranes‐on‐chip can be used for ion channel studies. a) Schematic of a chemical procedure of inducing membrane vesicle (bleb) formation as applied to the neuronal cells, following which the blebs are released and dispersed within the supernatant. b) Schematic showing the blebs deposited onto the PEDOT:PSS microelectrodes for formation of neuronal SLBs via vesicle‐fusion, as well as the EIS measurement setup (CE stands for counter electrode, and RE stands for reference electrode) for SLB characterisation and ion‐channel blocking studies. c) The actual chip consisting of four measuring compartments where the red‐square frame indicates the location of three electrodes within an eight‐electrode array. d) zoomed‐in image of the three electrodes in (c). The circular electrode is 450 µm in diameter, and the sides of the square electrode are each 200 µm in length. Scale bar, 200 µm.

Our approach to interface neuronal membranes leverages the biocompatibility of PEDOT:PSS thin films and its precise microfabrication into miniaturized electrodes (see **Figure** **1**c,d) to provide accurate and sensitive monitoring of the neuronal membrane using EIS. In the active electrode area, the thickness of PEDOT:PSS is ≈100 nm, and the 2 µm thick parylene C layer functions as insulation layer, inhibiting interactions between electrolyte and conductive tracks. As expected, the bigger (circular) electrode, with area four times the size of the smaller (square) electrode, exhibits lower baseline impedance compared to the smaller electrode (Figure S1, Supporting Information). For the native SLB formation, cell membrane vesicles are isolated based on the blebbing protocol presented by Sezgin and colleagues (Figure 1a).^[^ ^33^ ^]^ We adapted this protocol and optimised it for the SH‐SY5Y cells and have applied it before, as well as after, the 10‐day differentiation process. The resulting blebs are fused on the conducting polymer devices to form SLBs using the vesicle fusion method. As shown in previous work,^[^ ^11^, ^12^, ^15^, ^17^ ^]^ in order to facilitate fusion, fusogenic liposomes of 4:1 mixture of 1,2‐dioleoyl‐*sn*‐*glycero*‐3‐phosphocholine (DOPC) and 1,2‐dioleoyl‐3‐trimethylammonium‐propane (DOTAP) and polyethylene glycol (PEG8k), are added sequentially (Figure 1b). The formed SH‐SY5Y SLBs before and after differentiation were used for drug response tests with CaV ion‐channels.

### 2.1. SH‐SY5Y Differentiation

**Figure 2:**
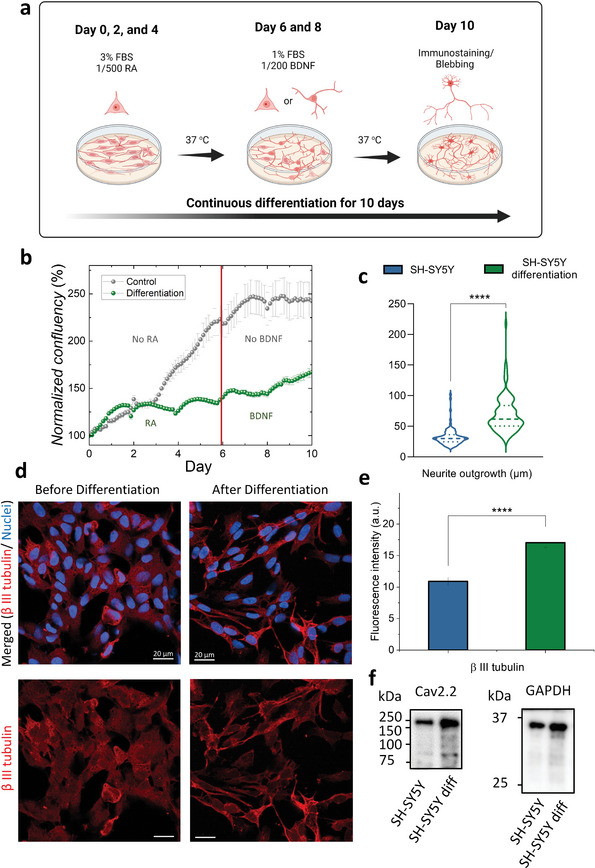
Phenotype and protein expression change in SH‐SY5Y cells after differentiation. a) Schematic showing the differentiation process of SH‐SY5Y cells: for day 0 to day 5, the cell‐culture media with 3% FBS and 1/500 RA was changed every other day; for day 6 to day 10, media with 1% FBS and 1/200 BDNF was changed every other day. The blebbing or immunostaining experiments were conducted on Day 10 as the endpoints. b) Comparison of cell confluency between the differentiation process (with adding media with RA (day 0–5) and BDNF (day 6–10)) and the control condition (with no RA and BDNF added). c) Box violin plots showing neurite outgrowth quantification (µm) of non‐differentiated and differentiated SH‐SY5Y cells. An unpaired Mann–Whitney U test was used to analyze differences between samples. Dash lines represent the median, and the dotted lines represent 75% and 25% of the data distribution, respectively. For both conditions (i.e., non‐differentiated and differentiated SH‐SY5Y cells), brightfield images of n = 6 cultures were used for analysis, and 89 and 90 neurites were manually traced for quantification, respectively. d) Fluorescence images of SH‐SY5Y cells morphology showing the changes in cell phenotype and biomarker expression before and after 10‐day continuous differentiation (red is β‐III‐tubulin; blue is Nuclei; Scale bar 20 µm). e) Average corrected fluorescence comparison of immunostained regions of β‐III‐tubulin signals (*n* = 10 areas of cells for non‐differentiated and differentiated cell cultures. The data points for each condition are tested for normal distribution and unpaired *t*‐test. The *p* ≤ 0.0001 is based on unpaired *t*‐test of these two conditions, where *, *p* ≤ 0.05, **, *p* ≤ 0.01, ***, *p* ≤ 0.001, ****, *p* ≤ 0). f) Western blot for comparing the expression level of CaV2.2 ion‐channels within the differentiated and non‐differentiated SH‐SY5Y cells.

Although SH‐SY5Y neuroblastoma cells are distinct from primary neurons, they naturally exhibit biochemical and functional similarity to human neurons and are widely used as a reliable in vitro neural model. The continuous treatment with RA and BDNF has been demonstrated to promote a more mature neuronal phenotype (e.g. neurite outgrowth, functional synapses).^[^ ^29^ ^]^ Continuously treated SH‐SY5Y cells also show increased expression of mature neural markers (e.g. microtubule‐associated protein 2 (MAP2) and heavy‐molecular‐weight neurofilament (NF‐H)) across the whole cell.^[^ ^30^ ^]^ Here, SH‐SY5Y cells undergo 5 days of differentiation with RA media followed by 5 days of treatment with BDNF media (see Experimental Section). The whole cell growth and differentiation process was recorded in the Video S1, Supporting Information, with a 3‐h time step for 10 days. For the first 5‐day window (day 0–5) of RA treatment, we observed an increased population of cells with branched extensions. Until day 6, the morphological population of the cells is a mixture of truncated cells without neurites (star‐shape) and cells with neurites (shooting‐star‐shape). Upon the addition of BDNF in the cell culture media (day 6–10) cells start developing longer neurite extensions enabling the establishment of neural networks with neighbouring cells (**Figure** **2**a). Furthermore, the addition of RA and BDNF seems to have an impact on reducing the cell proliferation speed. Compared to the control conditions (non‐differentiated SH‐SY5Y cells), which maintain the same media composition without adding RA or BDNF, the cell proliferation speed is much slower (Figure 2b). On day 10, the differentiated SH‐SY5Y cells are ≈78% less confluent than the control cells.

Neurite length (µm) of mature neurons is measured on images acquired from an Incucyte® live‐cell analysis system. As shown in the violin plots in Figure 2c, differentiated SH‐SY5Y cells exhibit longer axonal length, 69.9 ± 3.2 µm compared to the control non‐differentiated cells (32.3 ± 1.5 µm). Following the cell culturing steps, the differentiated or non‐differentiated SH‐SY5Y cells were fixed and immunostained for studying neuronal markers. Expression of β III tubulin, one of the most highly expressed neuronal markers within the cytoskeleton of matured neurons, was noticeably increased after the 10‐day differentiation (Figure 2d), and was quantified via immunofluorescence intensity estimation showing a significant increase after differentiation (Figure 2e). These results are consistent with the literature and our previous study of SH‐SY5Y differentiation on PEDOT:PSS‐based extracellular matrix (ECM) 3D scaffolds.^[^ ^29^, ^34^ ^]^ CaV2.2 ion channels, one of the most highly expressed ion channels on SH‐SY5Y, were found to have significant increased expression after the differentiation process as assessed via Western blot (Figure 2f). Within the differentiated and non‐differentiated SH‐SY5Y lysate, numerous types of proteins were present (See Coomassie blue stain in Figure S2a, Supporting Information). The band identifying the CaV2.2 ion channel (at ≈250 kDa) shows a more intense signal from the differentiated SH‐SY5Y cell sample compared to the non‐differentiated cells at the expected molecular weight (≈250 kDa).^[^ ^35^, ^36^ ^]^ Once the band intensities of CaV2.2 were normalized against the GAPDH, there is ≈ 92.2% increase of CaV2.2 signal after differentiation, which confirmed that the CaV2.2 ion channels were more upregulated during the differentiation process (raw images are shown in Figure S2b, Supporting Information).

### 2.2. Vesiculation Process and Characterization of the Blebs

**Figure 3:**
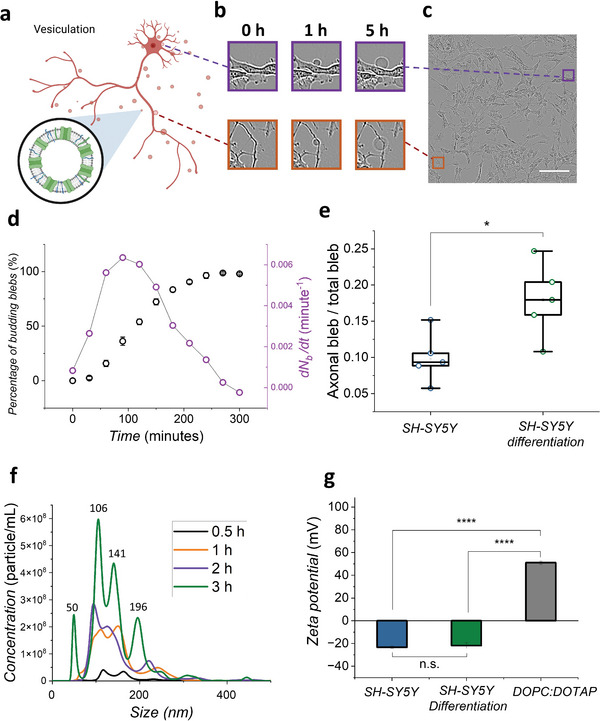
Vesiculation process and characterization of the blebs. a) Schematic of the blebbing process within vesiculation buffer. b) The time stamps of bleb size evolution from soma (purple) and axons (orange) in differentiated SH‐SY5Y culture at 0, 1, and 5 h time points (the whole process was recorded in Video S2, Supporting Information). c) The zoomed‐out bright field image of the experimental area (the dashed lines are pointing to the observed blebbing area in Figure b. scale bar, 100 µm). d) Percentage increase in SH‐SY5Y blebs (N_b_) over 300 min incubation under standard cell culture conditions (37 °C and 5% CO_2_). The first derivative of N_b_ with respect to time showing the rate of bleb formation (a representative plot is demonstrated). e) Ratio of axonal bleb population versus total bleb population from differentiated and non‐differentiated SH‐SY5Y cells at 90‐min time point (*n* = 5 for each condition. The data points from each condition are performed with normality test and unpaired *t*‐test. *p* = 0.0203 is based on unpaired *t*‐test comparing two conditions. This is analyzed from Incucyte images). f) The concentration profile of nano‐sized blebs from non‐differentiated SH‐SY5Y over 3 h (a representative plot is demonstrated). At the 3 h time point, the mean size of blebs is 145.6 ± 6.5 nm, and concentration is 1.0 × 10^9^ ± 3.14 × 10^8^ particle mL^−1^. g) Zeta potential of differentiated, non‐differentiated SH‐SY5Y blebs, and DOPC:DOTAP (4:1) liposomes. (*p* = 0.8117 is between SH‐SY5Y and SH‐SY5Y differentiation blebs. *p* < 0.0001 is for both SH‐SY5Y blebs and SH‐SY5Y blebs compared to DOPC:DOTAP liposomes). For (f,g), *, *p* ≤ 0.05, **, *p* ≤ 0.01, ***, *p* ≤ 0.001, ****, *p* ≤ 0.0001. n.s. stands for not significant.

As neuron‐like cells, SH‐SY5Y cells, especially following a differentiation process, have an inhomogeneous morphology (i.e., soma and axon) compared to other cells we have blebbed from (e.g. HEK293 cells), so the blebs from different parts of the cell membrane may be expected to have different protein content and expression level. It is crucial to have a deeper understanding of the origin of the blebs from SH‐SY5Y cell surfaces, which helps to explain biological events or interactions associated with transmembrane proteins. Therefore, we studied the blebbing process based on the protocol from Sezgin, et al.^[^ ^33^ ^]^ Due to the inhomogeneous nature of the neuronal cells, the blebbing process may yield a mixture of blebs derived from the axons and the soma with potentially different lipid and protein composition, in terms of amount and type. The vesiculation process was therefore monitored with an Incucyte® over 5 h (Video S2, Supporting Information). It was confirmed that the blebs were homogenously derived from the whole cell membrane without preference for particular cell structures (such as soma versus axons) (**Figure** **3**c). Single budding events of blebs from both the soma and axon are presented in Figure 3b. Through 5 h of recording of the blebbing process, we observed some micron‐sized blebs, increasing in size over the duration of recording. By studying the numbers of budding events, the number of blebs and the budding rate could be extracted (Figure 3d). Here, we define two characteristics of the blebbing process: time of end point of the blebbing process (t_end_) and time point of maximum budding rate (t_br,max_). t_end_ is the stopping point of the blebbing process, which shows 0 for the rate of blebbing. In the bleb population curve (Figure 3d), t_end_ is ≈270 min. The t_br,max_ of SH‐SY5Y happened at ≈130 min, which is featured by the highest point of the first derivative of bleb population with respect to the time (*d*N_b_/*d*t). For SH‐SY5Y, the blebbing characteristics (ex. t_end_ and t_br,max_) have similar values before and after differentiation. The population of axonal blebs compared to overall bleb number is increased after differentiation, as shown in Figure 3e, which is unsurprising given the increase in neurite length and number. The Incucyte® cannot give information on nanometer sized blebs, for which we turned to nanoparticle tracking analysis (NTA) (Figure 3f). The bleb solution was taken out for sampling at 30 min, 1, 2, and 3 h time points and assessed using NTA. The size groups (peaks at 106, 141, and 196 nm) of blebs were consistent over time, but the concentration of each size group shifted dramatically over 3 h. While the process of bleb formation can last for up to a few hours, it was observed that the similar average bleb size and range of size distribution with a little lower concentration could be obtained after only 2 h of blebbing (Figure S3, Supporting Information). Besides the sizes of the blebs, both differentiated and non‐differentiated blebs carry similar charge distributions which are −23.3 ± 1.1 and −21.8 ± 2.5 mV, respectively. This negatively charged membrane surface could have electrostatic interaction with drug molecules, so proper control experiments are necessary to confirm the changes of impedance spectra, which are induced purely from drug‐protein interactions. The native blebs from differentiated and non‐differentiated SH‐SY5Y cells have similar characteristics (e.g., size, concentration, and surface charge), which are promising prerequisites to reconstructing native cell membranes on supporting substrates or PEDOT:PSS devices.

### 2.3. Formation and quality of SH‐SY5Y SLBs

**Figure 4:**
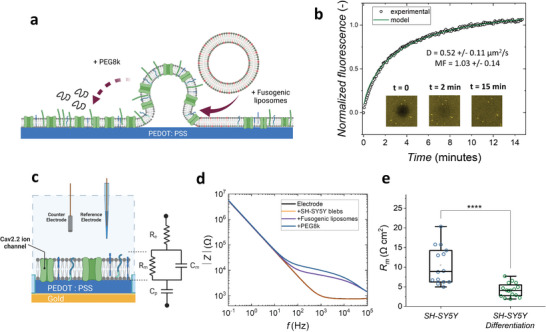
Monitoring of native SH‐SY5Y SLB with optical and electrochemical methods. a) Schematic showing the formation of a native SH‐SY5Y SLB via the step‐by‐step fusion method. b) Confirmation of lipid mobility within the differentiated SH‐SY5Y SLB using fluorescent recovery after photobleaching (FRAP) (D stands for diffusivity, and MF stands for mobile fraction. The inserts show the recovery of the bleached spot (30 µm in diameter) within 15 min). c) the setup of EIS measurement for the non‐differentiated and differentiated SH‐SY5Y SLBs. The model circuit mimics the electrochemical properties of the system (R_e_, R_m_, C_m_, and C_p_, are electrolyte resistance, membrane resistance, membrane capacitance, and PEDOT:PSS capacitance, respectively). d) Bode plot monitoring the impedance change of step‐by‐step formation of SH‐SY5Y SLB (a representative plot is demonstrated): 1) Bare electrode (black curve); 2) Addition of SH‐SY5Y blebs (orange curve); 3) Addition of fusogenic liposomes (DOPC:DOTAP, purple curve); 4) Addition of PEG8k (blue curve). e) The membrane quality is quantified by the membrane resistances (R_m_) of SH‐SY5Y (*n* = 14 electrodes are from 4 replicates of experiments with different batches of blebs and devices. Measurements are shown in blue circles) and differentiated SH‐SY5Y SLBs (*n* = 16 electrodes are from 4 replicates of experiments with different batches of blebs and devices. Measurements are shown in green circles. The data points from each condition are performed with normality and unpaired *t*‐test. *p* < 0.0001 based on the *t*‐test comparing membrane resistance of SH‐SY5Y and differentiated SH‐SY5Y, where *, *p* ≤ 0.05, **, *p* ≤ 0.01, ***, *p* ≤ 0.001, ****, *p* ≤ 0.0001).

SLBs were formed by first adding blebs onto the PEDOT:PSS devices, and fusogenic liposomes were added to trigger the rupture of the native SH‐SY5Y blebs (depicted in **Figure** **4**a).^[^ ^11^, ^12^, ^17^ ^]^ The addition of PEG (8000 kDa) solution promotes further “healing” of the SLB allowing for additional/remaining unruptured vesicles to rupture. One of the conventional methods to monitor and evaluate the properties of SLBs is fluorescence recovery after photobleaching (FRAP). The lipids within the SLB are labeled by lipophilic fluorescent molecules, such as octadecyl rhodamine B chloride (R18), and upon photobleaching of an area of the SLB, bleached fluorescence recovers by interdiffusion of bleached and unbleached lipids. By analyzing the recovery curve of the bleached area, figures of merit, diffusivity (D) and mobile fraction (MF) can be extracted to determine the fluidity of the SLB.^[^ ^37^ ^]^ Given the advantageous transparent nature of PEDOT:PSS films, FRAP can be performed on the PEDOT:PSS film for differentiated SH‐SY5Y SLB (Figure 4b) with excellent D = 0.52 ± 0.11 µm^2^ s^−1^ and MF = 1.03 ± 0.14, which is comparable to the literature values.^[^ ^15^ ^]^ These characteristics are crucial properties to mimic the cell membrane on PEDOT:PSS surfaces. Although FRAP provides a qualitative measure of SLB formation, monitoring SLBs formed on PEDOT:PSS coated microelectrodes with EIS is a more rapid and sensitive method to analyze the barrier‐effect of the membrane.

With our electrodes, we monitor the increase in “barrier‐effect” during the step‐by‐step fusion process while forming the SH‐SY5Y SLB using EIS, as parameters such as the resistance and capacitance of the membrane can be used to describe its characteristics, for example in terms of ion channel opening and closing. We previously presented a model circuit to depict the electrochemical properties of the membrane‐on‐a‐chip system (recapitulated in Figure 4c).^[^ ^11^, ^12^ ^]^ This step‐by‐step formation of native SLB was verified and quantified as shown in Figure 4d and Figure S4, Supporting Information. Incubation with fusogenic liposomes ruptured native blebs and caused the highest increase in impedance ≈351% (at 1000 Hz) with respect to the bare electrode impedance. Then, the PEG8k solution enhanced the barrier‐effect of the SLB (≈99% increase of impedance). The SLB was mimicked by a parallel circuit of membrane resistance (R_m_) and membrane capacitance (C_m_), in series with electrolyte resistance (R_e_) and PEDOT:PSS capacitance (C_p_). By fitting the frequency‐dependent EIS spectra, the R_m_ as a figure of merit could be applied to describe the ionic barrier‐effect of an SLB. For SH‐SY5Y SLBs, the R_m_ is similar to the literature values seen for other cells lines (i.e., HEK cells),^[^ ^17^ ^]^ and the R_m_ of differentiated SH‐SY5Y SLB (4.19 ± 0.43 Ω cm^2^) is around half of the non‐differentiated SH‐SY5Y R_m_ (10.50 ± 1.29 Ω cm^2^) (Figure 4e). We posit that this decrease of R_m_ after the differentiation process may be related to the higher‐level expression of membrane proteins so that the barrier effect of the SLB is reduced. This can be due to a higher number of open ion channels, or increased packing of proteins in the lipid bilayer which introduces defects.

### 2.4. Verapamil Interaction with CaV Ion‐Channel Monitored by EIS

Verapamil is a therapeutic molecule under the phenylalkylamine category of general calcium ion‐channel blockers.^[^ ^25^ ^]^ It has been clinically used to treat headaches and atrial arrhythmias.^[^ ^25^, ^38^ ^]^ Its blockage mechanism is completed by entering the pore of the CaV ion channel and specifically binding to the receptor site.^[^ ^25^ ^]^ Verapamil can bind to a wide range of CaV ion‐channel subtypes, such as CaV1.2, CaV1.3, CaV3.3, CaV3.1, CaV2.2, and CaV2.1.^[^ ^38^ ^]^ Therefore, it is an effective candidate molecule for neuronal CaV ion‐channel studies.

**Figure 5:**
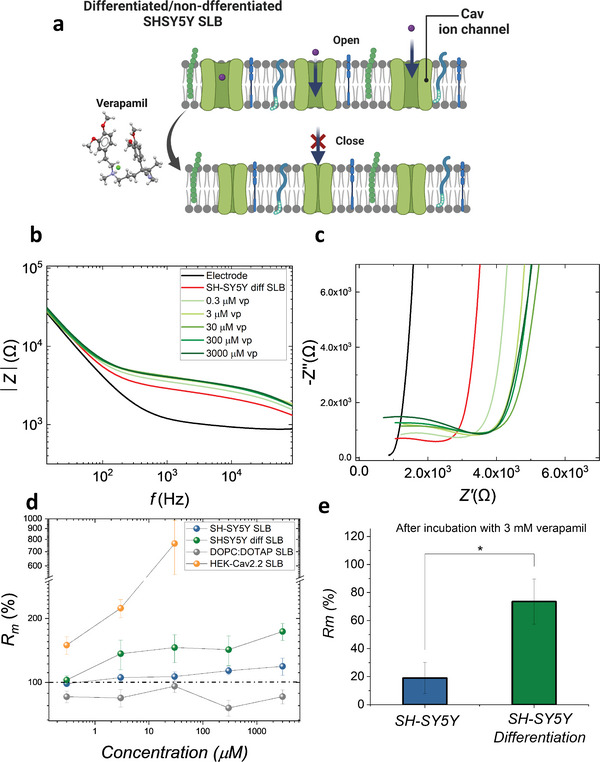
Increase blocking of calcium ion channel is seen in differentiated versus non‐differentiated SLBs. a) Schematics of verapamil molecules binding and blocking the CaV ion‐channel on the differentiated or non‐differentiated SH‐SY5Y SLB. b,c) The dose‐responsive EIS curves shows gradual increase of impedance when 0.3 µm to 3 mm of verapamil (vp) was sequentially added and incubated for 15 min (representative plots are demonstrated). b) Bode plot and c) Nyquist plot. d) The percentage R_m_ change of non‐differentiated SH‐SY5Y (*n* = 8 electrode measurements for each point) and differentiated SH‐SY5Y SLBs (*n* = 7 electrode measurements for each point) with positive (HEK cell SLB overexpressed with CaV2.2 ion‐channels) and negative controls (all lipid SLB (DOPC:DOTAP)). e) Percentage increase after addition of 3 mm verapamil was incubated with both non‐differentiated and differentiated SH‐SY5Y SLBs (The data points from each condition are performed with normality test and unpaired *t*‐test. The *p* = 0.0133 based on *t*‐test comparing these two conditions, where *, *p* ≤ 0.05, **, *p* ≤ 0.01, ***, *p* ≤ 0.001, ****, *p* ≤ 0.0001).

We performed verapamil dose‐response studies on differentiated and non‐differentiated SH‐SY5Y SLBs and monitored its effect using our membrane‐on‐a‐chip system (see schematic in **Figure** **5**a). As shown in Figure 5b,c, upon interaction with verapamil, both the differentiated and non‐differentiated SH‐SY5Y SLBs EIS spectra show an increase in the recorded impedance, most likely attributed to the blocking of the CaV ion channels. In the literature, the concentration of verapamil from 0.3 to 10 µm has shown a gradual depressing effect to the calcium ion currents, so we selected 0.3 to 30 µm for testing the sensitivity of the system as well as higher concentrations (300 µm and 3 mm) to see the saturation effect of blocking ion channels.^[^ ^39^, ^40^ ^]^ Low concentrations of verapamil (0.3, 3, and 30 µm) brought only a subtle increase in the R_m_ of the non‐differentiated SLB and a more significant increase of R_m_ for concentrations from 300 µm up to 3 mm (Figure 5d). The observed changes of the impedance were more pronounced in the differentiated SH‐SY5Y SLBs. Even at the lowest concentrations (0.3 and 3 µm), a response was sensed, indicated by a bigger semi‐circles in the Nyquist plot compared to the baseline (red curve) of the membrane (Figure 5c,d). Further incubation of higher doses caused more blocking effects. Once we added the highest concentration (3 mm), the blocking‐effect was saturated as expected, with an average of around 2.9  times R_m_ increase in differentiated SH‐SY5Y SLBs (73.5% ± 16.1% increase) detected compared to non‐differentiated SLBs (19.0% ± 11.1% increase). This is very likely related to the higher expression of CaV ion channels after the differentiation of SH‐SY5Y cells, which was confirmed with Western blot in Figure 2. Besides SH‐SY5Y SLBs, SLBs were also formed from blebs derived from human embryonic kidney cells which overexpressed the CaV2.2 ion channels (HEK‐CaV2.2) as well as from synthetic liposomes (DOPC:DOTAP) only, which were tested as positive and negative controls, respectively. Since HEK‐CaV2.2 overexpressed CaV2.2, the drug response was more pronounced. More than a ≈700% increase in R_m_ was sensed at only 30 µm of verapamil whereas in the case of DOPC:DOTAP SLBs where the protein is not present, no increase in membrane resistance was observed (tothe slight decrease in membrane resistance resides from artifacts introduced by the washing). The increased blocking‐effect of CaV ion channels on SH‐SH5Y SLBs matches the live cell results showing increased CaV ionic current after a process of differentiation presented by Toseli, et al.^[^ ^32^ ^]^

### 2.5. Verapamil Blocking Assay of CaV Ion‐Channels Performed on Primary Cortical Neuronal SLBs

**Figure 6:**
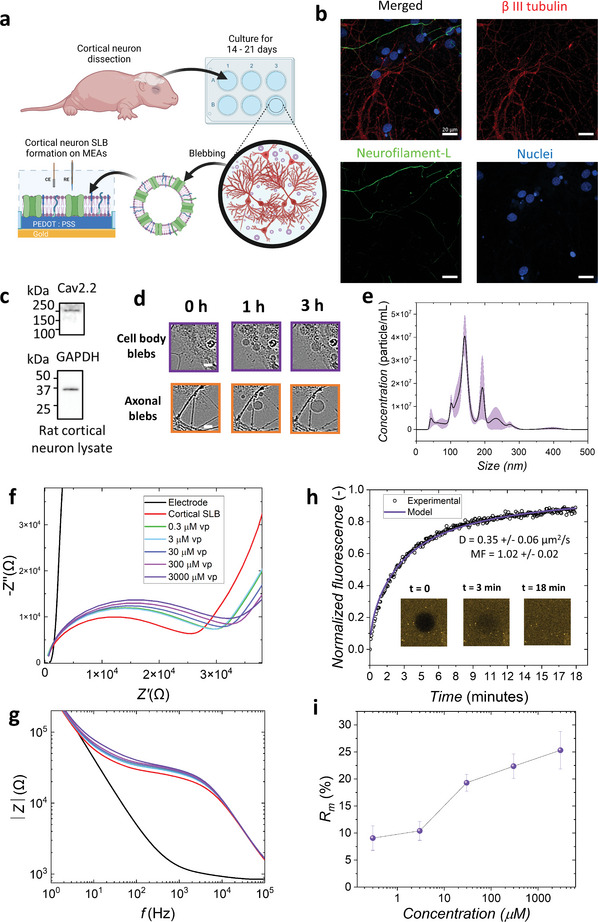
SLBs from primary cortical neurons can be used for calcium ion channel studies. a) Schematic of isolation of neuronal blebs and SLB formation from rat cortical neuron primary culture for dose response experiments. First, the primary cortical neurons are isolated from post‐natal day 1 rats. Second, the primary cortical cells are cultured for 14–21 days for neuron maturation. With the blebbing processes, the primary cortical neuronal blebs are induced and then the SLBs are formed. b) Immunostaining images for cortical neuron culture after 21 days. (red: β III tubulin; green: neurofilament‐L; blue: nuclei. scale bar, 20 µm). c) Western blot shows CaV2.2 ion‐channels and GAPDH present indicated by the band at ≈250 and ≈36 kDa markers, respectively. d) Real‐time tracking (0, 1, and 3 h) of blebbing formation process from cortical neurons with demonstration of blebs from neuron cell body (purple) and axon (orange). Scale bar 15 µm. e) NTA measurements of nano‐scale blebs from cortical neurons with 154.9 ± 11.2 nm average size and 4.14 × 10^8^ ± 5.67 × 10^6^ paricles mL^−1^ (*n* = 3). f, g) Representative EIS plots of dose‐response of verapamil binding and blocking on cortical neuronal SLBs: f) Nyquist plot; g) Blode plot. h) FRAP of cortical neuronal SLB on PEDOT:PSS thin films with diffusivity (D) 0.35 ± 0.06 µm^2^ s^−1^ and mobile fraction (MF) 1.02 ± 0.02 (*n* = 3). i) Dose‐response of Rm percentage increase of verapamil on cortical neuronal SLBs (*n* = 10 for each data point).

To prove the concept of studying voltage‐gated ion‐channel blocking effects for more typical neuroscience research, we progressed to make SLBs from primary cortical neurons (PCN) and then to monitoring of verapamil blocking activity with CaV ion‐channels on this membrane‐on‐a‐chip system. As illustrated in **Figure** **6**a, the primary cortical neurons are dissected and isolated from brains of post‐natal day 1 rats. Then, the cortical neurons need to be maintained in standard cell culture environment for 14–21 days until mature. Through this process, the neurons spread and extend axons to form neuronal networks with neighbouring cells, which is a well‐established method for in vitro primary neuron studies.^[^ ^41^ ^]^ The blebs are isolated from the cortical neurons at this stage. Then, the PCN SLB integration is achieved by the vesicle fusion method as described above for SH‐SY5Y SLBs.

After 14–21 days of culture, the PCNs show healthy and matured status, with the topographically web‐like axonal network being one of the crucial indicators (Figure 6b). Also, typical matured neuron‐markers, β III tubulin and neurofilament‐L, are significantly expressed in the cytoskeleton of the cells. Western blot was also performed and CaV2.2 expression further confirmed by a clear band at ≈250 kDa. Further, the blebbing process was characterised with Incucyte® and NTA. Formation of the micro‐sized blebs was monitored for 3 h continuously by Incucyte® (Video S3, Supporting Information). The blebbing process from PCNs has a similar behaviour to the blebs from differentiated SH‐SY5Y cells. Blebs come from the whole cell including cell body (soma) and axons (Figure 6d). However, the observed population of micron‐sized blebs is much more than from the SH‐SY5Y cells, so manual counting of population for extraction of t_end_ and t_br,mas_ values is not feasible. Bleb samples are usually filtered through 450 nm pore filters, so the cell debris and giant blebs (micro‐sized blebs) are eliminated. NTA determined that the average concentration of the nano‐sized blebs was 4.14 × 10^8^ ± 5.67 × 10^6^ particles mL^−1^. As Figure 6e demonstrates, the dominant peak of nano‐sized blebs is ≈150 nm, and sub‐100 nm and 200 nm blebs are shown as lower‐concentration peaks.

As the cortical‐neuron blebs have comparable characteristics in terms of size and concentration to SH‐SY5Y blebs, high‐quality PCN SLBs can be integrated on PEDOT:PSS thin‐film surfaces. With FRAP, excellent membrane mobility was characterized and quantified by diffusivity (D = 0.35 ± 0.06 µm^2^ s^−1^) and mobile fraction (MF = 1.02 ± 0.02) matching the standard values in the literature of other native SLBs on PEDOT:PSS films.^[^ ^15^ ^]^ By EIS, the PCN SLB demonstrated a high membrane resistance of 35.11 ± 2.57 Ω cm^2^ (Figure S5, Supporting Information). Just like the verapamil dose‐response experiment on SH‐SY5Y SLBs, the same test was also performed on PCN SLBs, and PCN SLBs demonstrated a step‐by‐step ion‐channel blocking behavior at each dose. When verapamil was sequentially tested (from 0.3 µm to 3 mm), the width and the height of the SLB semi‐circle (Nyquist plot in Figure 6f) both increase, which indicates the CaV ion‐channel blocking effects are induced by verapamil. From the Bode plot (Figure 6g), the plateau at mid‐frequency range (10–4000 Hz) dominates the increase on the spectra, and the impedances in the high frequency range (4000 Hz and above) perfectly overlap at each dose. This indicates that the R_m_ increase is the major effect on the membrane, which is caused by the binding/blocking effect of verapamil with the CaV ion channel.^[^ ^17^ ^]^ Figure 6i depicts the increasing manner of R_m_ with each concentration of verapamil. At 0.3 and 3 µm, the R_m_ increase is ≈10%, and then 30 µm triggers the highest total increase (≈19%). Literature has demonstrated that verapamil suppresses slow CaV current ≈0.2 to 8 µm with patch clamp techniques,^[^ ^40^, ^42^ ^]^ which matches the low‐concentration range associated with the CaV ion‐channel blocking effect observed with this system. The highest two concentrations (300 µm and 3 mm) further increase the R_m_, but with saturation effects. The cortical SLB has ≈25.3 ± 3.5% increase of R_m_ at 3 mm (Figure 6i). Compared with SH‐SY5Y SLBs, cortical neuron SLBs have an R_m_ with a similar final increase to non‐differentiated SH‐SY5Y, although interestingly, the average R_m_ increase of differentiated SH‐SY5Y is still significantly higher than that of the cortical neuron SLBs (Figure S5b, Supporting Information).

## 3 Conclusion

We have demonstrated that microfabricated organic microelectrode arrays can be integrated with native neuronal membranes and employed as a platform for rapid dose‐response testing with neuronal ion‐channel blocking drugs. Since this system interfaces with the native neuronal membrane, it preserves the activity of native transmembrane ion‐channel proteins and can avoid many technical difficulties of maintaining live cells and trial and error processes, such as patch clamp. In contrast to the traditional patch clamp method, the neuronal‐membrane‐on‐a‐chip approach is a cell‐free, time‐efficient, and high‐throughput technique.

Testing this platform with SH‐SY5Y before and after differentiation as well as primary neurons from rats shows the versatility of this system for interfacing different cell types. With this neuronal‐membrane‐on‐a‐chip system, we demonstrated: 1. The possibility of forming native SLBs derived from both neuron‐like cells and primary neurons; 2. The increased expression of CaV ion channels (focusing on CaV2.2) on differentiated SH‐SY5Y membranes compared to the non‐differentiated ones by electrical monitoring; 3. Dose‐response of CaV ion‐channel blocking effect on primary cortical neuronal SLBs. The dose range causing ion channel blocking is comparable to results from literature.^[^ ^40^, ^42^ ^]^ Surprisingly, the SLBs made from cortical neurons showed lower verapamil response than the differentiated SH‐SY5Y blebs. This may however illustrate the challenge inherent in using cell lines where calibrating the concentration of ion channel to the native cell level is difficult.

In addition, we have studied and optimized the blebbing condition for formation of high quality SH‐SY5Y SLBs and defined the time of end point of the blebbing process (t_end_) and time point of maximum blebbing rate (t_max,br_) as figures of merits of this process. For different cell types and different blebbing conditions (formulation of the vesiculation buffer and temperature), these values could vary. Therefore, following the goal of forming high quality SLB, the blebbing process should be optimised for each cell type. The t_end_ and t_br,max_ extraction and analysis currently rely on manually counting; the same analysis is not feasible for blebs from cortical neurons due to high population of microscopic blebs. One limitation here however, is that the counting is done on the micron sized blebs and there may be a divergence in population between the micron and nanometer sized particles. Further, since the blebs are derived from across the whole neuronal cell membrane, rather than a localised patch as would be the case for patch clamp, the blebs presumably give an average representation of the whole cell membrane. Last but not least, membrane resistance (R_m_) is a universal value to depict cell membrane quality. This parameter is the key characteristic to quantify the drug efficacy in this work. However, the R_m_ from reconstructed native SLBs is generally a much lower value compared to the value obtained from patch clamp on live cells. This is due to giga‐Ohm seals formed between the patch clamp pipette and the cell. However, the challenge in patching a cell remains a major obstacle. In planar patch clamp systems, giga‐Ohm sealshave been achieved however the limitation there is the inability to patch neurons.^[^ ^6^, ^7^ ^]^ Future work using our platform will focus on increasing the R_m_ from SLBs. We believe this is currently constrained by a number of factors: bleb properties (ex. concentration, size distribution), expression of native transmembrane protein, PEDOT:PSS surface (ex. surface charge and roughness). Optimisation of these properties will form the directions for future advancement of this technology.

To facilitate the application of these organic MEAs for interfacing neuronal membranes as a regular neurology research tool, the neuronal SLB should have high yield of formation and representative native components. There are clear directions to optimise for this system: eliminating the use of non‐native lipids (e.g., DOTAP) for fusogenic liposomes, functionalization of electrode surfaces can also be used for improving vesicle fusion,^[^ ^43^ ^]^ concentration of blebs, and authenticity of transmembrane proteins. The concentration of blebs appears to play an important role of native domains on the SLB.^[^ ^44^ ^]^ This could be adjusted by optimisation of the formulation of the blebbing buffer. Furthermore, the authenticity of the transmembrane proteins dictates the efficiency of the bio‐events and sensitivity of the platform, so chemical concentration (formaldehyde (FA) could denature the native protein over time) and blebbing time could be readjusted for the best performance of the native proteins.

Neuronal‐membrane‐on‐a‐chip platforms are not only a drug screening system to test blockage effects of ion channels as shown here, but could also be used in future as a platform to study neurological phenomena and diseases (such as the causes of neurodegenerative effects related to Parkinson's and Alzheimer's disease (AD)). Not only due to aberrant signaling of ion channels, but also due to the interaction of species such as amyloid peptides and Tau with lipids, implicated in AD. SLBs formed from disease model animals could allow high‐throughput screening of future therapeutics as a means of evaluating interactions at the molecular level. This system could accelerate the preliminary drug screening process for selecting brain‐related treatments by mimicking native neuronal membranes as a high‐throughput complementary technique to more expensive, challenging, and time‐consuming in vivo tests.

## 4 Experimental Section

### OMEA Fabrication

The microelectrodes used, shown in Figure 1, were circular electrodes with 450 µm diameter and square electrode with 200 µm for each side. The dimensions of the electrodes were designed for optimal SLB sensitivity, which was discussed in the previous work.^[^ ^17^ ^]^ To fabricate the devices, 4 inch glass wafers were first cleaned by sonication in acetone and then isopropanol for 15 min. The wafers were rinsed with DI water and baked 15 min at 150 °C. To pattern for contact tracks, a negative photoresist, AZ nLOF2035 (Microchemicals GmbH) was spun on the glass wafer with 3000 rpm for 45 s and exposed with UV light using a mask aligner (Karl Suss MA/BA6). The photoresist was developed in AZ 726 MIF developer (MIcroChemicals) developer for 28 s. Ti (5 nm)/Au (100 nm) layer was deposited by e‐beam evaporation on top of wafer and the Ti‐Au metal layer was lifted‐off by soaking in NI555 (Microchemicals GmbH) overnight for patterning the conductive tracks. Prior to the deposition of 2 µm layer (sacrificial layer) of parylene C ((SCS), the wafer was soaked with 3% A174 (3‐(trimethoxysilyl)propyl methacrylate) in ethanol solution (0.1% acetic acid in ethanol) 60 s to promote the parylene C adhesion on the wafer. An anti‐adhesive layer of Micro‐90 in DI water (2% v/v solution) was spun (1000 rpm for 45 s), and then the second layer of 2 µm parylene C (SCS) was deposited. A layer of positive photoresist AZ 10XT (Microchemicals GmbH) was spun with 3000 rpm for 45 s and developed in AZ 726 MIF developer (MIcroChemicals) for 6 min to pattern electrode areas. Reactive ion etching (Oxford 80 Plasmalab plus) opened the window for deposition of Clevios PH500 PEDOT:PSS (Heraeus). At this point, the wafer can be cut into chips with 4 working arrays (Figure 1c). The PEDOT:PSS solution containing 5 vol% ethylene glycol, 0.26 vol% dodecylbenzenesulfonic acid (DBSA), and 1 vol% (3‐glycidyloxypropyl)trimethyloxy‐silane (GOPS) was spin‐coated on to each chip at 3000 rpm for 45s. The chips were baked at 90 °C for 1 min, and the sacrificial layer was peeled off. Finally, the chips were put on a hot plate at 130 °C for 1 h before use.

### SH‐SY5Y Cell Culture

SH‐SY5Ycells were maintained in 1:1 Minimum Essential Media (MEM; Sigma Aldrich) and Nutrient Mixture F‐12 Ham (Sigma‐Aldrich) with 15% fetal bovine serum (FBS, Sigma‐Aldrich), 1% non‐essential amino acids (NEAA 100X, Sigma Aldrich), 1% Glutamax 1–100X (Life Technologies) and 1% Antibiotic‐Antimycotic. Splitting was performed at 70–80% confluency and medium refreshed every 3–4 days. In brief, after overnight incubation at 37 °C in 75 cm^2^ flasks, cells were washed with 5 mL Dulbecco's phosphate saline (DPBS) (Sigma‐Aldrich), then incubated with 2.5 mL 0.25%Trypsin‐EDTA (Invitrogen) for 4 min at 37 °C, 5% CO2, and finally, they were resuspended in 6 mL of fresh culture medium to stop trypsinization. Upon centrifugation at 1000 rpm × 5 min, the medium was aspirated and the pellet was resuspended in fresh culture medium, counted in a hemocytometer, and seeded in new T‐75 flasks with a final cell density of 1 × 10^4^ cells cm^−2^.

### SH‐SY5Y Differentiation

The differentiation protocol was performed based on previous studies,^[^ ^34^, ^45^, ^46^ ^]^ with slight modifications: Briefly, 2 × 10^4^ cells cm^−2^ SH‐SY5Y were seeded in each well of 6‐well plate with or without poly‐L‐lysine (Sigma) pre‐coated glass coverslips in 15% FBS culture media. Until 70% confluency (day 0), cells were washed once with DPBS, and cell media were replaced with 3% FBS culture media +10 µm retinoic acid (RA, Sigma‐Aldrich) and changed every other day (day 2 and day 4). On day 6, cells were washed once with DPBS and incubated with 1% FBS culture media containing Neurobasal medium (Life Technologies) supplemented with B‐27 (Thermo Fisher) and 50 ng mL^−1^ brain‐derived neurotrophic factor (BDNF, Thermo Fisher). Media change occurred every other day (day 8), and cells were fixed after 10 days of differentiation for further blebbing process or imaging analysis. Neurite outgrowth analysis was performed using the semi‐automated ImageJ plugin, NeuronJ.

### Primary Cell Culture

Cortical tissues were isolated from postnatal day 1 rats (Sprague–Dawley rats from Charles River) and digested in Dulbecco's modified eagle medium (DMEM, Thermo Fischer Scientific) containing 0.1% Trypsin and 0.05% DNAase (Sigma–Aldrich) for 20 min in an incubator. The tissues were dissociated to single cell suspension by trituration through 1 mL and 200 µL pipette tips and the suspension was centrifuged at 600 rpm for 5 min. The supernatant was replaced by Neurobasal medium containing 2% B27 and 0.25% Glutamax (all from Thermo Fisher Scientific) and the cell pellet was gently resuspended. Finally, cells were plated on a 6 well‐plate (Nunc, Thermo Scientific) at a cell density of 50,000–200,000 cells, previously coated with poly‐L‐lysine (Sigma). The cultures were maintained for 2–3 weeks by replacing half of the medium once a week.

### Cell Fixation and Immunostaining:

Cells on glass coverslips were fixed with 4% PFA in Phosphate buffered saline (PBS) for 5 min at room temperature. Samples were permeabilized in DPBS with 0.25% Triton X‐100 for 10 min and then blocked with 3% BSA + 0.1% Tween‐20 in PBS for 30 min both at room temperature, after washing the samples three times with PBS, samples were incubated with β III tubulin monoclonal antibody eFluor660 (5 µg mL^−1^, Thermo Fisher) and CaV 2.2 antibody (CACNA1B (N type) Polyclonal Antibody, Alomone Labs, ACC‐002. According to the product page from Alomone Labs, this antibody works for CaV2.2 proteins both from human and rat) 1:125 dilution ratio overnight at 4 °C, the samples were washed three times with DPBS and incubated for 1 h with Goat anti‐Rabbit IgG (H+L) Cross‐Adsorbed Secondary Antibody, Alexa Fluor 488 (1:500, Thermo Fisher) and then incubated with Hoechst (10 ug mL^−1^, Abcam) for 15mins. All images were acquired using a confocal microscope (Axio Observer Z1 LSM 800, Zeiss) with the detector gain value of 650 V for all channels.

### Cell Lysate Preparation

The lysing buffer was prepared by mixing protease inhibitor cocktail and EDTA with RIPA buffer with 1:1:198 ratio. The lysate buffer was added to the cells at 52.1 µL cm^−2^ and was incubated with cells for 5 min. The supernatant was collected and centrifuged with 13 000 rpms ^−1^ speed for 15 min. The supernatant was collected and frozen at −80 °C.

### Western Blot and Coomassie Stain

The total protein quantification was conducted by using Qubit^TM^ Protein Assay Kits and following the provided protocol. The Qubit^TM^ working solution was prepared by mixing Qubit^TM^ Protein Reagent and Qubit Protein Buffer with 1:200 ratio. The cell lysate samples were taken out and defrosted at room temperature. Then, each lysate sample needed to be diluted 10 times, and then 1 µL of diluted lysate was added into 199 µL Qubit^TM^ working solution. The concentration of the total protein was determined by Qubit 3.0 Fluorometer.

The undiluted lysate sample was first mixed with sample buffer (2× Laemmli sample buffer (BIO‐RAD catalog #161‐0737) with 100 mm DTT) with 1:1 ratio. Then, each sample was heated at 96 °C for 5 min. The electrophoresis was performed using a Mini‐PROTEAN System (BIO‐RAD). Each sample was loaded to each well with 40 µg of total protein, and electrophoresis was run at 200 V for 35 min. For Coomassie stain, the gel needed to be soaked in Coomassie blue dye for 1 h, and protein bands were shown after more than 1 h of soaking within water.

For Western blot, the protein lanes within the gel was transferred onto a PVDF membrane with semi‐dry transfer process (BIO‐RAD) with 15 V for 20 min. The transferred PVDF was soaked with blocking buffer (3% BSA and 0.1% Tween‐20 in PBS) for at least 1 h. The PVDF is soaked and shaken over night with primary antibody solution (CaV 2.2 antibody (CACNA1B (N type) Polyclonal Antibody, Alomone Labs, ACC‐002): blocking buffer = 1:200; GAPDH antibody: blocking buffer = 1:500) overnight at 4 °C. On the second day, secondary antibody solution (secondary antibody: blocking buffer = 1:5000) was soaked and shaken for 1 h. The membrane needed to be incubated within chemiluminescent reagents (SuperSignal West Pico PLUS Chemiluminescent Substrate) for 1 min in the dark. Last, the membrane was quickly transferred for imaging (G:Box, Syngene).

### Blebbing from Differentiated, Non‐Differentiated SH‐SY5Y Cells, and Primary Cortical Neurons

This protocol was adapted from previous studies.^[^ ^47^, ^48^ ^]^ The cells were differentiated or cultured in 6‐well plates or T75 flasks (Thermal fisher) at 37 °C and 5% CO_2_. The cells were washed with GPMV buffer (2 mm CaCl_2_, 10 mm HEPES, 150 mm NaCl at pH 7.4) one time. Then, the cells were incubated with blebbing buffer which consisted of GPMV buffer with 25 mm formaldehyde (FA) and 2mM dithiothereitol (DTT). For general extraction of membrane vesicle, the incubation time is ≈2 h. For the study of blebbing process, the incubation was tested over 3–5 h. The supernatant containing blebs was collected and placed in ice in a tube for 15 min, which helps to separate the cell debris from the blebs. The top 85 vol% of the solution with bleb was carefully taken for further experiments.

### NTA for Bleb Sizes and Concentrations 

The concentration and size distribution of blebs isolated from non‐differentiated SH‐SY5Y cells, differentiated SH‐SY5Y cells, and primary cortical neurons were determined by Nanoparticle tracking analysis (NTA, Nanosight NS500, Malvern), with samples diluted in PBS to obtain 20–40 particles/frame. The dilution ratio was multiplied back to obtain the concentration spectra for the final plot.

For the study of bleb profiles from SH‐SY5Y cells, the SH‐SY5Y cells were first cultured in a T75 flask until ≈80% confluence. Following the same blebbing protocol shown above, the blebbing buffer was incubated with cells over 3 h. Bleb samples were taken at time points of 0.5, 1, 2, and 3 h. Each sample was then filtered through a 450 nm filter. NTA measurements were taken for filtered bleb samples following dilution with PBS to maintain 20–40 particles/frame.

### Zeta Surface Potential of Blebs

Measurements (*n* = 3) were performed on Malvern Zetasizer Nano. A total 1 mL volume of diluted sample was used for in Zetasizer cuvette for zeta surface potential measurements. 100 µL of bleb sample was mixed with 900 µL of milli‐Q water.

### Preparation of Synthetic Liposomes

The 1,2‐dioleoyl‐*sn‐glycero*‐3‐phosphocholine (DOPC) and mixed with 1,2‐dioleoyl‐3‐trimethylammonium‐propane (DOTAP) lipid (Avanti Polar Lipids) in chloroform (25 mg mL^−1^) was used for the experiments. DOPC was mixed with DOTAP with 4:1 volumetric ratio and dried under nitrogen gas. The lipid mix was further dried under vacuum at room temperature for 1 h, to evaporate the residual chloroform. PBS buffer was added to rehydrate the lipids to a concentration of 4 mg mL^−1^. The solution was frozen at −20 °C for at least 5 h. Then, the defrosted solution was extruded 20 times through a 100 nm membrane (GE Healthcare).

### Formation of SH‐SY5Y SLBs

Prior to formation of the SLBs, the PEDOT:PSS surface either on a glass slide or on an electrode was soaked with 70% ethanol for at least 30 min to clean the residue from fabrication. After the PEDOT:PSS‐coated surface was exposed with air plasma (Harrick Plasma, Ithaca, NY at 18 W), 100 µL of SH‐SY5Y blebs were quickly added onto the surface and incubated for 20 min and then washed with PBS (three times). The fusogenic liposomes (DOPC:DOTAP 4 mg mL^−1^ in PBS) were added and incubated for 20 min with further rinsing with PBS (three times). A 8000 kDa poly(ethylene glycol) solution in PBS (PEG8k, 30% w/v) was further added and incubated for another 20 min. Finally, the formed SLBs needed to be rinsed with PBS five times before further experiments.

### FRAP Experiments

The experiments were performed on an inverted Zeiss LSM800 confocal microscope (Zeiss Germany) with a 10× objective. To label the membrane, 1 µL of 0.36 mm octadecyl rhodamine B chloride (R18, Molecular Probes) fluorophore was added to 200 µL of bleb or liposome solution in a soft sonication bath for 15 min. Excess fluorophore was removed by using a G25 spin column (GE Healthcare). Starting from the labelled blebs, supported lipid bilayers were assembled as described. A 150 mW 561 nm optically pumped semiconductor laser (Coherent, Inc.) was used to photobleach a 30 µm diameter spot in the supported lipid bilayer and its fluorescence intensity recovery was monitored up to 15–20 min. The fluorescence intensity change over time was fitted using a Bessel function, following the method of Soumpasis.^[^ ^37^ ^]^ The diffusion coefficient was calculated with the following equation:

(1) $$D=w^{2}/4t_{1/2}$$

where *w* is the radius of the photobleached spot and *t*_1/2_ is the time required to achieve half of the maximum recovery intensity.

### EIS

Commercial Ag/AgCl and a homemade platinum‐mesh electrode were used as reference and counter electrodes, respectively. The micro‐fabricated PEDOT:PSS coated Au microelectrodes as the working electrodes. An Autolab PGSTAT128N potentiostat equipped with a frequency response analyzer was used to record impedance spectra in the frequency range between 1Hz (or 0.1 Hz) and 100 kHz. The working, reference, and counter electrodes are connected to the corresponding channels of the potentiostat. An AC voltage of 0.01 V and a DC voltage of 0 V versus OCP (open circuit potential) were applied. All measurements were taken in ≈200 mL PBS retained on the chip by a glass well.

### CaV Ion‐Channel Blocking Essay with Verapamil

This process was conducted on non‐differentiated/non‐differentiated SH‐SY5Y SLBs and primary cortical neuron SLBs. The EIS spectra of electrodes were obtained prior to the formation of SLBs. After formation of the SLBs, the SLBs were incubated at room temperature for at least 1 h for lipid stabilization, and EIS was measured before the drug tests. A concentration series (0.3, 3, 30, 300, and 3000 µm) of verapamil was prepared in PBS by serial dilution. During the experiment, the concentrations were added from low to high. Before adding the testing concentration, 150 µL of testing concentration was first added to wash the well. Then, add another 150 µL testing concentration and incubation at room temperature for 15 min. Each SLB‐on‐electrode needed to be washed with PBS two times before the EIS measurement.

### Ethical Protocol

Work was approved by the Animal Welfare and Ethical Review Body (AWERB) of the University of Cambridge and by the UK Home Office (approval number: NR2023/20). AWERB was responsible for housing and maintaining all animals used in these studies. This ensured that the animals received ethical treatment and were in compliance with the guidelines and regulations governing animal research. The primary cortical neurons for all the experiments (including immunostaining, Western blot, blebbing, and verapamil tests on the biomembrane‐on‐a‐chip system) were from two different dissections. Postnatal day 1 rats were dissected and the cortical neurons were isolated from each session.

## Conflict of Interest

The authors declare no conflict of interest.

## Author Contributions

Z.L. and R.M.O. conceptualised the whole project and the experiments. Z.L. and A.S. designed the MEA. Z.L. microfabrication of the PEDOT:PSS‐coated MEAs. C.B. established the SH‐SY5Y cell differentiation protocol. The Differentiation of SH‐SY5Y cells was conducted by Z.L. A.F.V. contributed to the cortical primary neuron dissection, isolation, and culture. C.B. and A.W. maintained the non‐differentiated SH‐SY5Y cells. B.M.H. provided HEK‐Cav2.2 cells. A.W. and Z.L. contributed to the immunostaining and confocal imaging. Z.L. also contributed to the study of monitoring of SH‐SY5Y differentiation and blebbing process with Incucyte. Z.L. conducted Western blots, zeta surface potential measurements. A.W. measured NTA for the blebs. Z.L. and E.M. formed first non‐differentiated SH‐SY5Y SLBs on PEDOT:PSS surfaces. Z.L. further formed differentiated SH‐SY5Y and cortical neuron SLBs PEDOT:PSS surfaces and characterised the three types of SLBs with EIS and FRAP. Z.L. performed verapamil dose‐response experiments on non‐differentiated and differentiated SH‐SY5Y as well as cortical neuron SLBs. Z.L. and K.K. performed the dose‐response experiment on HEK‐CaV2.2 SLBs. Z.L. contributed to data plotting, data analysis, biostatistics, and arrangement of the figures, as well as created the schematics for each figure. C.B. conducted the neurite outgrowth analysis. The first draft and last draft were written and checked by Z.L., and edited by all the co‐authors. The project was supervised by R.M.O., A.M.P., and G.S.K.

## Supporting information

Supporting Information

Supporting Information

Supporting Information

Supporting Information

## Data Availability

The data that support the findings of this study are available from the corresponding author upon reasonable request.;
