# Supplementary material for: Microelectrode Arrays Measure Blocking of Voltage‐Gated Calcium Ion Channels on Supported Lipid Bilayers Derived from Primary Neurons
Source: Adv Sci (Weinh). 2023 Dec 1;11(27):2304301. doi: 10.1002/advs.202304301 (PMC11251556; doi:10.1002/advs.202304301)
Supplement: Supplementary file 1 — Supporting Information [file ADVS-11-2304301-s003.pdf]

ADVANCED SCIENCE
Open Access

Supporting Information

for *Adv. Sci.*, DOI 10.1002/advs.202304301

Microelectrode Arrays Measure Blocking of Voltage-Gated Calcium Ion Channels on Supported Lipid Bilayers Derived from Primary Neurons

*Zixuan Lu, Chiara Barberio, Ana Fernandez-Villegas, Aimee Withers, Alexandra Wheeler, Konstantinos Kallitsis, Eleonora Martinelli, Achilleas Savva, Becky M. Hess, Anna-Maria Pappa, Gabriele S. Kaminski Schierle and Róisín M. Owens**

Supplementary information

Microelectrode Arrays Measure blocking of Voltage-Gated Calcium-ion Channels on Supported Lipid bilayers Derived from Primary Neurons

*Zixuan Lu, Chiara Barberio, Ana Fernandez-Villegas, Aimee Withers, Alexandra Wheeler, Konstantinos Kallitsis, Eleonora Martinelli, Achilleas Savva, Becky M. Hess, Anna-Maria Pappa, Gabriele S. Kaminski Schierle and Róisín M. Owen**

Table of Content

Supplementary Video 1-3 captions

Supplementary Figures S1-S5

Supplementary Videos 1-3

Video 1 | Video of recording 10-day sequential differentiation process as described in Figure 2a. The axon growth and branching were captured every 3 hours. Retinoic acid (RA) differentiation media was applied for day 0-5, and brain-derived neurotrophic factor (BDNF) media was applied for day 6-10.

Video 2 | This video recorded the blebbing process over 5 hours shown in Figure 3b. The population and size of membrane vesicles (blebs) were observed with scale bar, 200 μm.

Video 3| This video recorded the blebbing process over 3 hours shown in Figure 6d. The population and size of membrane vesicles (blebs) were observed with scale bar, 15 μm.

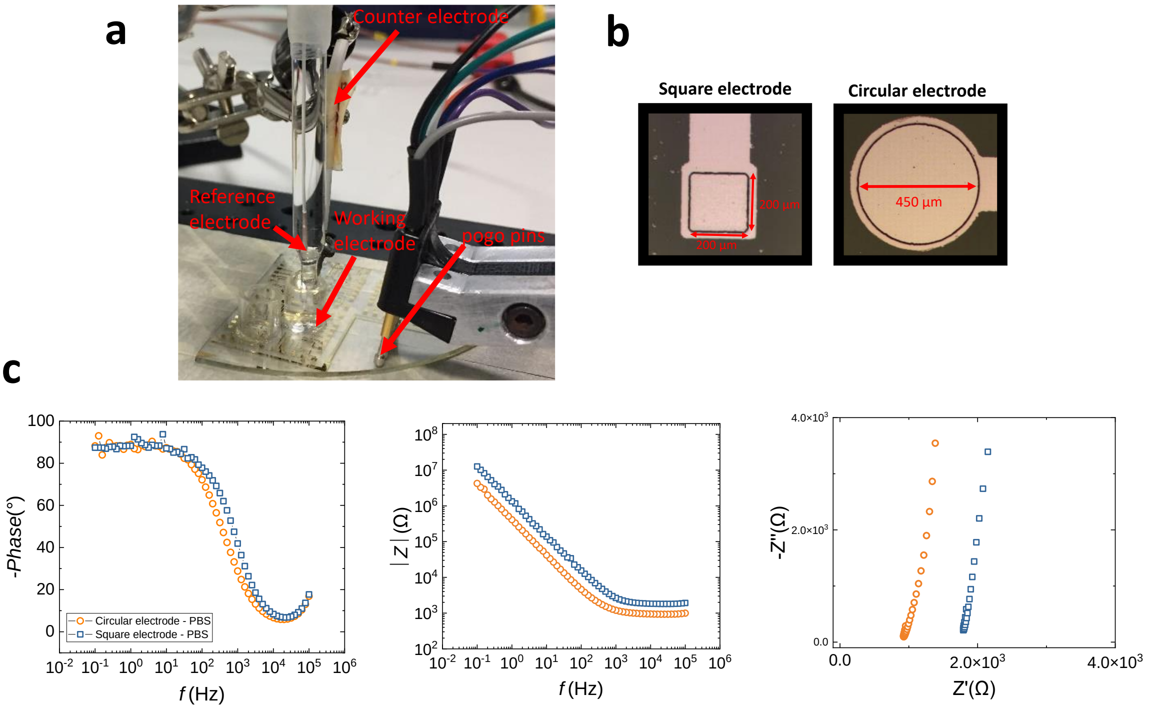

Figure S1. Microelectrode dimensions and characteristics electrochemical impedance spectroscopy (EIS). a, The setup of EIS measurements. b, The dimensions of square and circular electrodes. c, The EIS spectra of square and circular electrodes (representative curves): Phase of Bode plot (left); Impedance of Bode plot (middle); Nyquist plot (right).

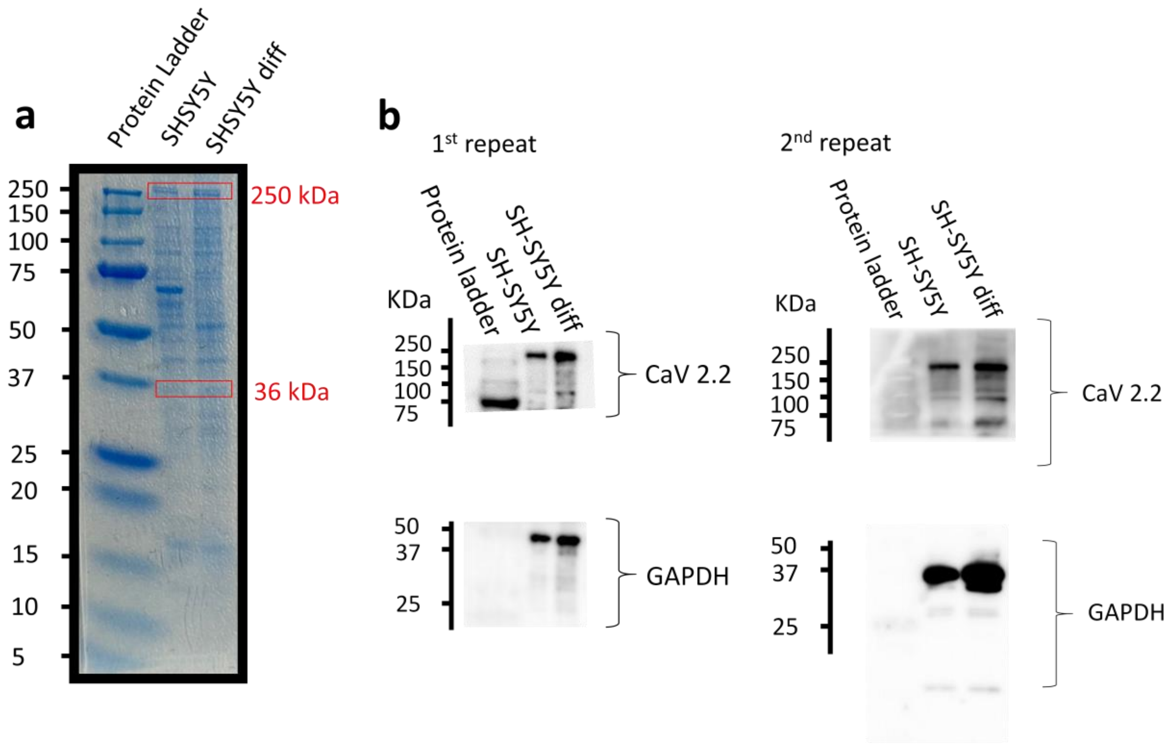

Figure S2. Coomassie blue stain and original images of Western blot stain with differentiated and non-differentiated SH-SY5Y cell lysate. a, The protein bands for Cav2.2 (~ 250 kDa) and GAPDH (~36 kDa) are labeled with red boxes. a, Two replicates of Western blot images of anti-CaV2.2 stained top-half membrane (250-75 KDa) and anti GAPDH stained bottom-half membrane (50-25 KDa).

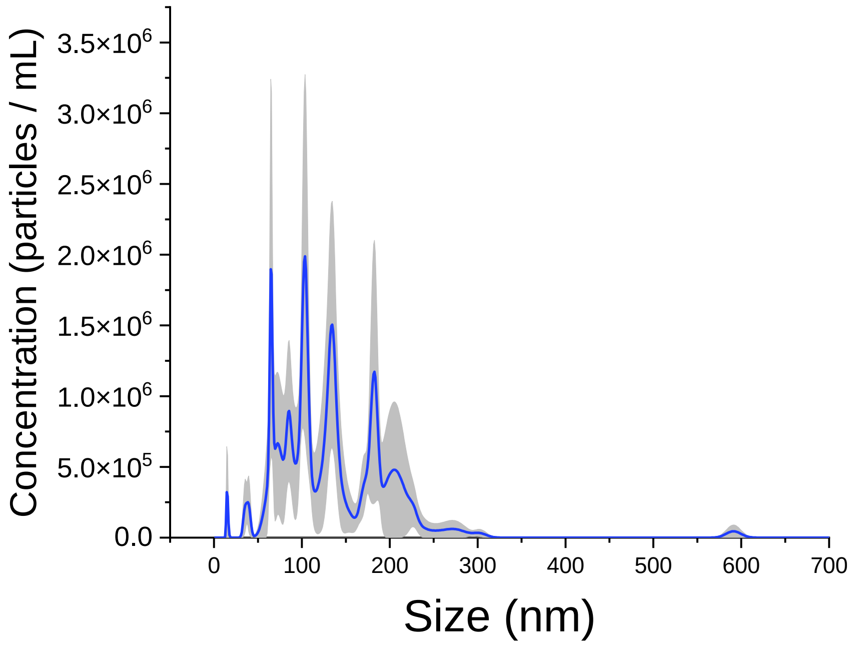

Figure S3. Nanoparticle tracking analysis (NTA) after 2 hours of blebbing. The resulting concentration distribution with respect to blebs sizes of non-differentiated SH-SY5Y blebs with 2 hour incubation (n = 3). The mean size is 135 ± 13.9 nm, and the concentration is 1.8 $\times 10^8 \pm 4.93 \times 10^7$ particle/mL

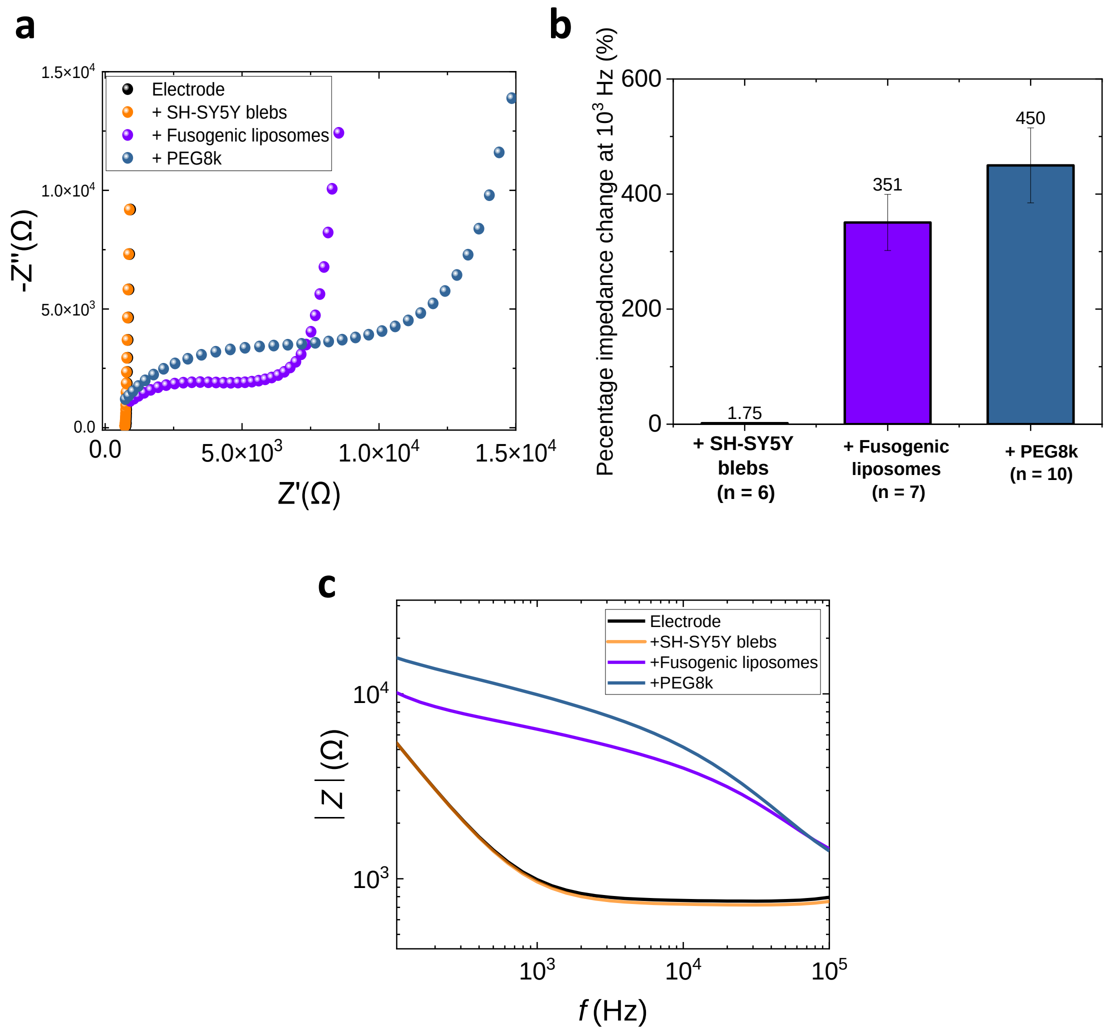

Figure S4. The step-by-step fusion process monitored by EIS. a, The Nyquist plot shows the increase in size of the semicircles at each step of vesicle fusion indicating an increase in resistance. b, The percentage increase of impedance at 10^3 Hz after each step of vesicle fusion. c, The zoomed-in plot of Figure 4d (Bode plot).

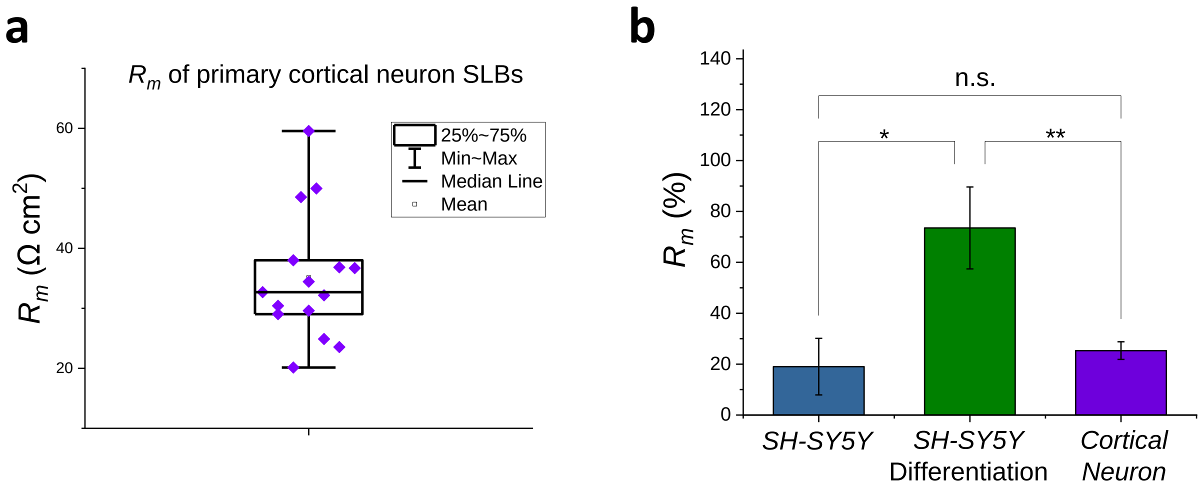

Figure S5. Average R_m of primary cortical neuron SLBs: a, box plot and data points of R_m of primary cortical neuron SLBs. The mean value is 35.11 ± 2.57 Ω cm^2 (each SLB R_m is displayed as purple dots. n = 15). b, Bar chart to compare the final R_m increases of cortical neuron SLBs (n = 10 SLB measurements from 2 separated experiments) after adding 3mM verapamil with the R_m data of SH-SY5Y, differentiated SH-SY5Y in Fig. 5 (normality test and unpaired t-test were performed on each pair of conditions. The p = 0.48 is between SH-SY5Y and cortical neuron SLBs. The p = 0.0023 is between SH-SY5Y differentiation and cortical neuron SLBs. *, $p \leq 0.05$, **, $p \leq 0.01$, ***, $p \leq 0.001$, ****, $p \leq 0.0001$. n.s. stands for not significant).
